# Supplementary material for: Speaker-normalized sound representations in the human auditory cortex
Source: Nat Commun. 2019 Jun 5;10:2465. doi: 10.1038/s41467-019-10365-z (PMC6549175; doi:10.1038/s41467-019-10365-z)
Supplement: Supplementary file 1 — Supplementary information [file 41467_2019_10365_MOESM1_ESM.pdf]

Supporting information to:

Speaker-normalized sound representations in the human auditory cortex

Sjerps et al.,

SUPPLEMENTARY FIGURES

S1:

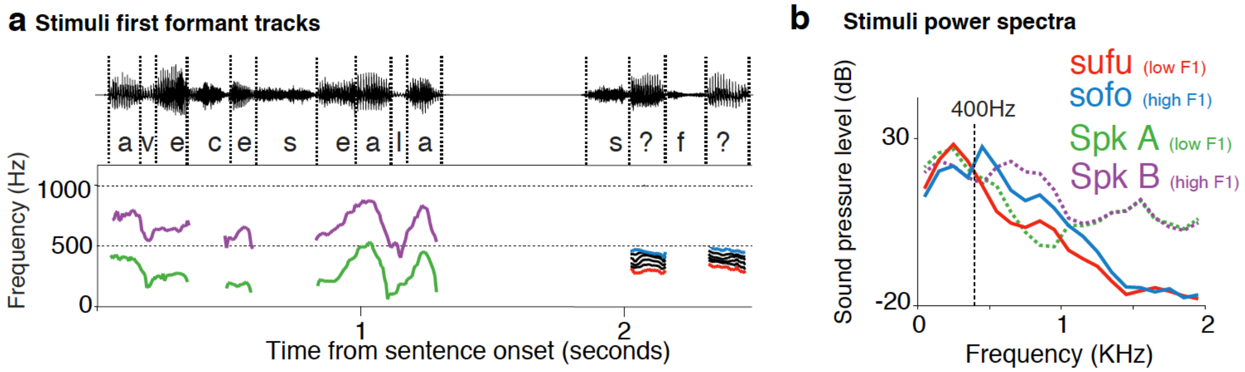

*Figure S1: **Contexts and targets display similar spectral differences**. **a**) First formant tracks for the voiced portions of the synthesized materials. In the annotation "?" indicates one of the target vowel steps. **b)** A similar spectral relation exists between the two endpoint targets /u/ vs. /o/ and the context sentences. /u/ and the low F1 speaker have more dominant low frequency components (i.e., below 400 Hz) in the spectrum than /o/ and the High F1 speaker.*

S2:

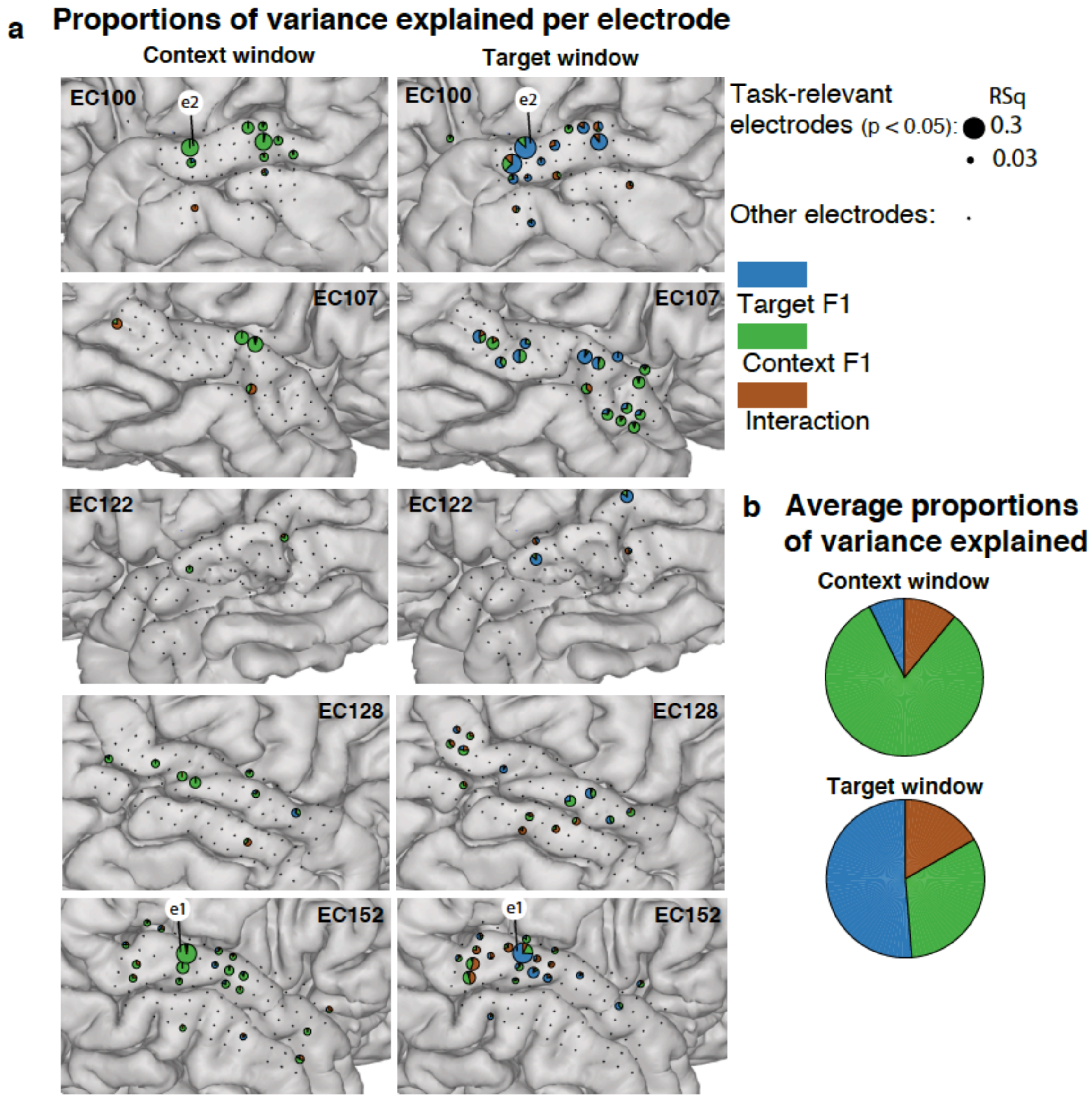

Figure S2: ***Context F1 influences cortical activity during target processing. a****) Map of context and target encoding for all task-related electrodes of all subjects (temporal lobe only), both during the context window (left column) and during the target window (right column). The areas of the pie-charts are proportional to the total variance explained. Wedges show the relative variance explained by each factor (stimulus dimension) for each significant electrode.* ***b****) Weighted average proportion of variance explained by main effects and interactions across all significant electrodes (across all 5 patients). During the context window, context properties explain the large majority of variance. During the target window, the context stimulus properties still explain a considerable portion of the variance.*

S3:

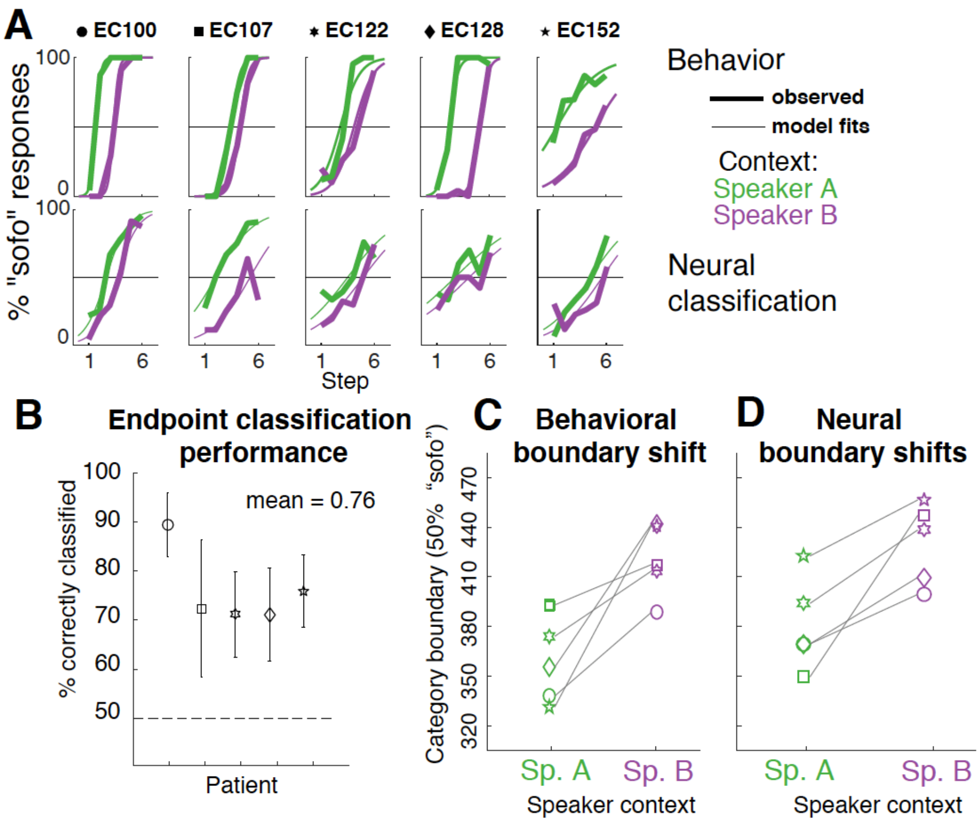

Figure S3: ***All participants display context effects in both their behavior and neural responses****.* ***a****) Top row: Observed mean proportions of "sofo" responses across the steps of the continuum in both context conditions for all participants (thick lines). Model fits (thin lines) are used to estimate the 50% category boundary per condition per participant (used for panel c). Bottom: same as in top panel but for the neural classification data (thick lines reflect LDA-based predictions).* ***b****) overall percentage correct (leave-one-out) classification on the endpoints per participant (with bootstrapped 95% CI).* ***c****) By-participant indications of the estimated 50% category boundaries in the two context conditions based on behavior.* ***d****) Same as c but for 50% category boundary estimates of the neurally-based classification (i.e., the data is identical to Figure 2d, but is reproduced here for comparison to Fig. S3c). Symbols denote individual participants.*

S4:

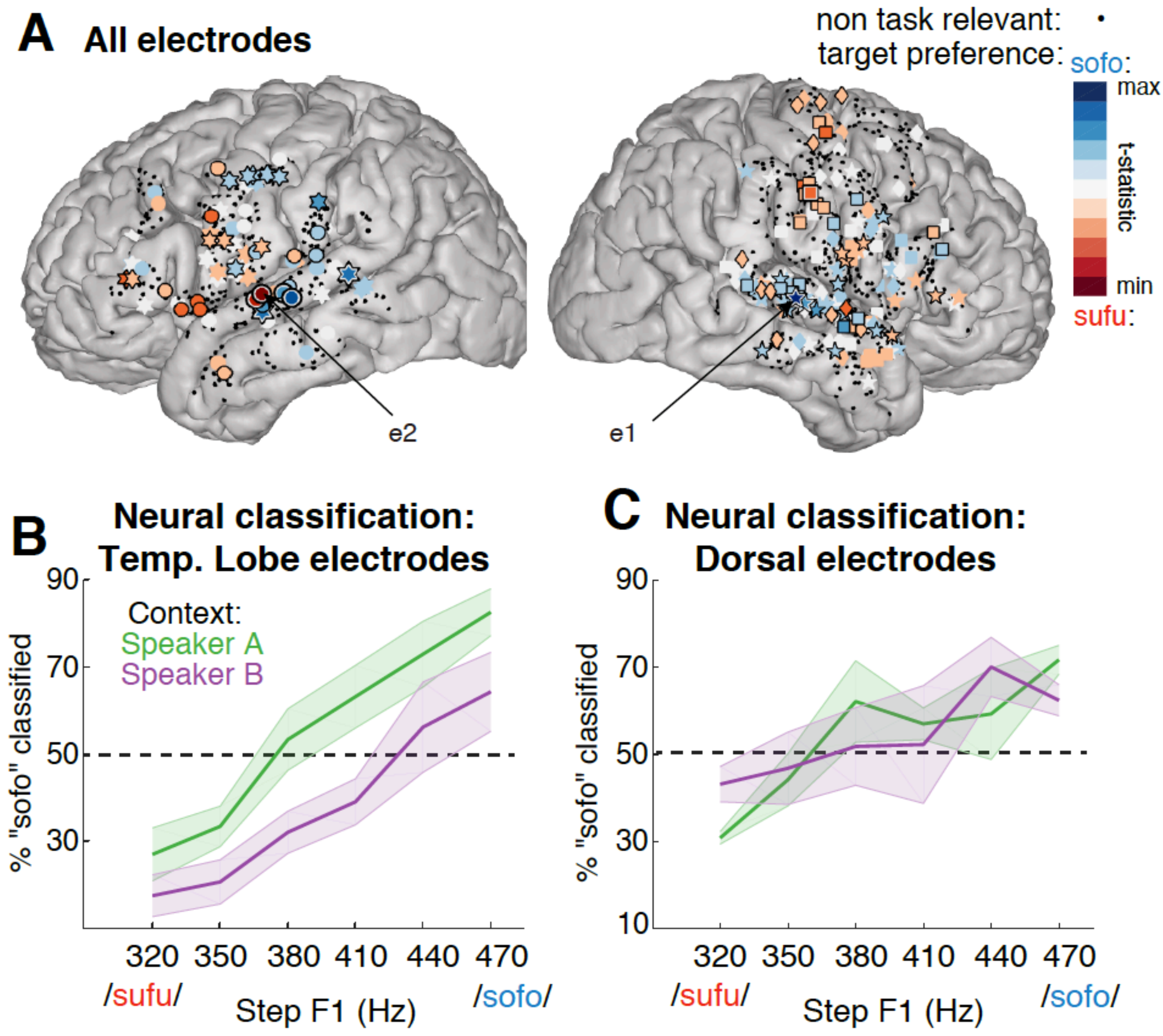

*Figure S4**: Normalization is observed only in temporal lobe regions a**) Target vowel selective electrodes are congregated on the temporal lobe, although some are also found in frontal regions. Symbols denote individual participants (see S3 for reference). **b**) when including temporal electrodes, LDA classification results (averaged across participants) reveal a strong context effect. **c**) no context effect is observed for LDA classification results based on electrodes from dorsal (i.e., all non-temporal) regions. In b and c, error bars indicate +-1 s.e.m.*

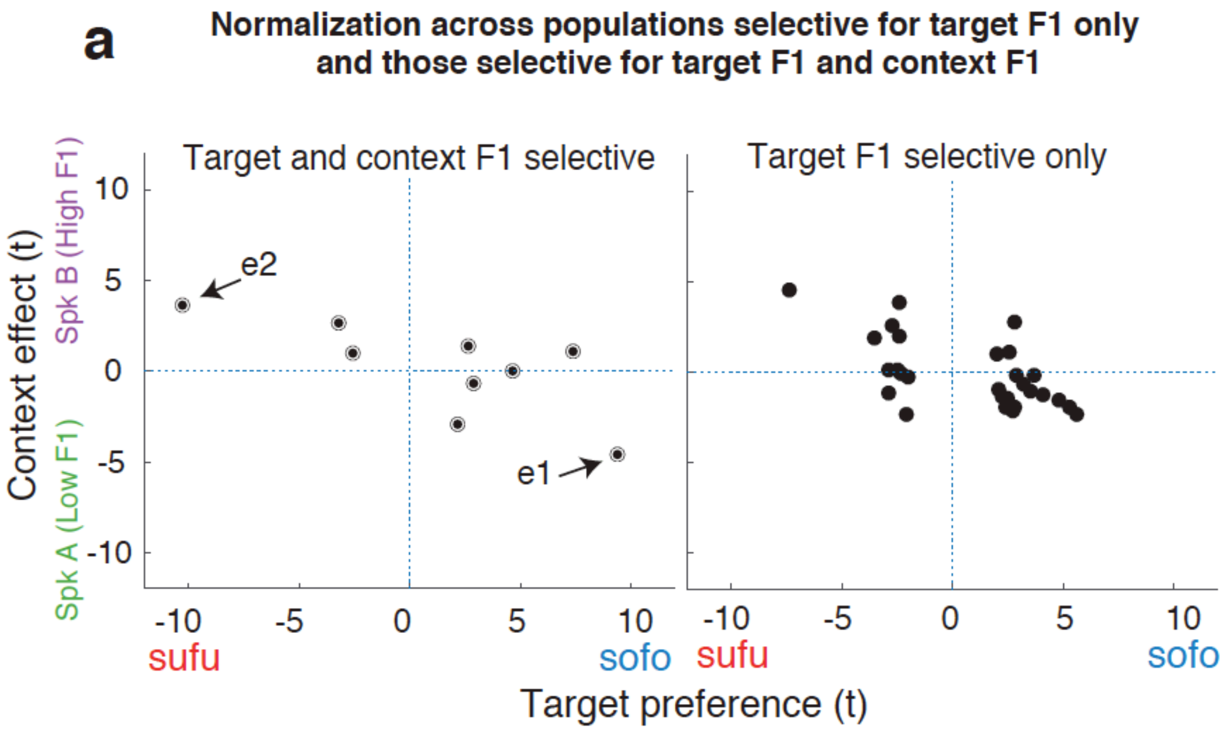

Figure S5: ***Normalization across target F1 selective population types****. Panels displays the same relation as Figure 2d, (the relation between target preference and context effect; i.e., normalization), but for two types of populations. Normalization is observed for electrodes that are most clearly selective for acoustic-phonetic features (instead of phonemes) since they display preferences for both target F1 and context F1 (left panel; black-and-white outline; target and context materials contain different phonemes). For completeness; normalization is also observed for those electrodes that display target F1 preferences only (right panel; black fill). See Figure 3 for reference.*

SUPPLEMENTARY TABLES

Table 1: **Trial counts**

Item	EC100	EC107	EC122	EC128	EC152
High_1	24	9	28	23	33
High_2	24	9	26	23	26
High_3	24	12	31	24	31
High_4	23	11	27	24	27
High_5	24	11	29	19	29
High_6	24	9	27	22	26
Low_1	23	11	25	21	31
Low_2	24	12	27	21	29
Low_3	24	9	23	20	33
Low_4	23	8	30	20	31
Low_5	24	10	25	19	31
Low_6	23	11	28	21	31

Table 2: **Electrode type counts** (temporal lobe)

Patient	Task-related	Target F1(corrected)	Context F1(corrected)
EC100	19	10(4)	8(3)
EC107	19	7(1)	6(2)
EC122	7	3(2)	3(0)
EC128	21	7(0)	10(2)
EC152	32	11(2)	16(4)
